# Development, coinfection, and the syndemics of pregnancy in Sub-Saharan Africa

**DOI:** 10.1186/2049-9957-2-26

**Published:** 2013-11-15

**Authors:** Merrill Singer

**Affiliations:** 1 Department of Anthropology and Department of Community Medicine, University of Connecticut, Storrs, CT 06269, USA

**Keywords:** Syndemics, Coinfection, Maternal health, Sub-Saharan Africa, Millennium development goals

## Abstract

Notable among gaps in the achievement of the global health Millennium Development Goals (MDG) are shortcomings in addressing maternal health, an issue addressed in the fifth MDG. This shortfall is particularly acute in Sub-Saharan Africa (SSA), where over half of all maternal deaths occur each year. While there is not as yet a comprehensive understanding of the biological and social causes of maternal death in SSA, it is evident that poverty, gendered economic marginalization, social disruptions, hindered access to care, unevenness in the quality of care, illegal and clandestine abortions, and infections are all critical factors. Beyond these factors, this paper presents a review of the existing literature on maternal health in SSA to argue that syndemics constitute a significant additional source of maternal morbidity and mortality in the region. Increasing focus on the nature, prevention, and treatment of syndemics, as a result, should be part and parcel of improving maternal health in SSA.

## Multilingual abstracts

Please see Additional file 1 for translations of the abstract into the six official working languages of the United Nations.

## Review

### Quandaries in health development and pregnancy in Sub-Saharan Africa

As 2015 draws near, Gorman [1] highlights the fact that although there has been notable progress in several critical areas of global health, one of the Millennium Development Goals (MDGs) adopted at the 2000 Millennium Summit “has been particularly recalcitrant to progress… namely, improving maternal health.” The risk of a woman dying as a result of pregnancy or childbirth is about one in six in the poorest nations of the world compared with about one in 30,000 in the wealthy nations of Northern Europe [2]. The United Nations Secretariat [3] estimated that almost 300,000 maternal deaths occurred in 2010, 56% of them in Sub-Saharan Africa (SSA). With a maternal mortality rate of 640 maternal deaths per 100,000 live births [4], achieving MDG-5 in SSA is proving to be a formidable challenge. More broadly, maternal survival has been a comparatively neglected area in global health [5,6], suggestive of the longstanding primacy of focusing on fetuses and children, and not mothers, in maternal and child health programming [7]. Reflective of this shortcoming, as Gorman affirms, there is not as yet a full understanding of the biological and social causes of maternal death in SSA, although it is clear that poverty, gendered economic marginalization, social disruptions, access to care, quality of care, illegal and clandestine abortions, and infections are all critical factors [8-10]. The question has been raised: is achieving MDG-5 an impossible dream [11]?

Pregnancy is a unique immune condition that often has been characterized as a highly risky state for the mother and fetus alike. As Mor and Cardenas [12] note, for example, “Pregnant women in malaria-endemic regions are more susceptible to malaria infection during the first half of the pregnancy and this risk gradually declines during the second half. Lassa fever, caused by infection with an arenavirus showed a higher rate of case fatality in pregnant women particularly in the third trimester”. Additionally, pregnancy may increase susceptibility and increase rates of mortality due to various infection diseases [13].

Compared to other world regions, infection as a cause of maternal death is especially prominent in SSA [14]. While it is evident that vulnerability to infection during pregnancy is conditioned by various factors, including the stage of the pregnancy, the nature of the disease challenge, the health of the mother prior to becoming pregnant, and access to quality health care, most existing discussions consider the immune challenge mounted by a single infectious agent. But what of the ability of the immune systems of pregnant women to respond to comorbid disease challenges, including diseases that are known to interact adversely in dually or multiply infected individuals? Moreover, what of the role of reciprocal actions during pregnancy involving the deleterious interface of infectious and noninfectious conditions? Adverse disease interactions, or syndemics, have been shown in recent years to play a significant detrimental role in the health of vulnerable populations [15].

The purpose of this paper is to emphasize the potential degree of influence of syndemics based on a review of an uneven, in many ways limited, but nonetheless suggestive, literature on the health of pregnant women in SSA. Focusing specifically on women’s health, I argue that among the issues that must be addressed to a greater extent in health development efforts in the region are the emergence of an increased and more systematic awareness of, and response to, the syndemics of pregnancy.

#### The biosocial model of syndemics

The predominant biomedical and epidemiological models of disease stress isolated focus on individual threats to health. Such models rest on three underlying assumptions: every case of illness has a single underlying cause, a specific and identifiable disease is the source of each illness, and removal or reduction of the disease will produce a return to health [16]. Yet, as Valderas *et al.*[17] stress, on a daily basis biomedical practitioners treat “individuals with multiple coexisting diseases, who are now the norm rather the exception”. That the great majority of patients, as well as sufferers not in care, have more than one health problem at any given moment needs to be explicitly acknowledged if the health impacts of disease complexity are to be effectively addressed.

Feinstein [18] introduced the concept of comorbidity to label the co-occurrence of diseases or other disorders. It has become increasingly apparent how important this concept is in health. A study of comorbidity in Canada, for example, concluded that findings on the high prevalence of multiple conditions “call into question the very organization of our health services [19].” In SSA, it is likely that the significance of comorbidity for population health and individual patient treatment is several magnitudes greater than in Canada, creating what Levitt *et al.*[20] term a health “collision course”: “Noncommunicable diseases … are emerging, and their risk factors are becoming more common as lifestyles change and rates of urbanization increase. Simultaneously, epidemics of infectious diseases persist, and HIV/AIDS has taken hold in the region”. Indeed, it has been suggested that SSA is in the midst of a profound health transition involving a quadruple burden of infectious, noninfectious, injury-related, and perinatal and maternal health problems [21]. As a result, Starfield [22], who believes focus should not be on individual threads but on the “tapestry of morbidity,” suggests that patients “should be characterized by their morbidity burden and, more importantly, by the patterns of morbidity that they experience with time”.

With an understanding of the force of comorbidity in health, a syndemics orientation draws attention to two critical issues. First, diseases can transition beyond copresence to consequential adverse interaction. Second, the clustering of diseases in populations and the enhanced vulnerability of particular groups is often a result of social conditions and the unequal structure of social relationships. The differences between the terms comorbid and syndemic, as Mustanski *et al.* stress [23], is not simply semantic. Research guided by a comorbidity model tends to focus on the disease boundaries, overlaps, and prioritization, while syndemic research directs attention to “communities experiencing co-occurring epidemics that additively increase negative health consequences [23]”. The adverse synergistic interaction of diseases in syndemics, in other words, multiplies the burden of disease in a population, and, under given conditions, can escalate contagion, disease progression, disability, and mortality. Stall *et al.*[24] introduced the syndemic production model, which posits that the greater the number of conditions in a syndemic interaction, the more adverse the health outcomes.

Vulnerability to syndemics involves both factors that put groups in harm’s way for clusters of disease, as well as factors that contribute to the weakening of bodies, the degrading of immune capacities, the failing of social support systems, and the disruption or inaccessibility of healthcare services. Commonly, these are social conditions, such as structural inequalities, which produce chronic stress, inadequate diets, exposure to physical and emotional traumas, gender or other discrimination, and involvement in risky behaviors such as survival or coping mechanisms [15,25]. These kinds of social encumbrances “get under the skin” and have direct impact not only on disease development and progression, but on deleterious disease interaction as well.

Syndemics, in short, involve both biological and social factors and their pathways of interaction, mechanisms of disease promotion, and enhanced health impacts. Syndemic theory draws attention to, and provides a framework for, the analysis of these biosocial interactions, including their causes and consequences for human life and well-being [26-28]. Syndemics research has examined the entwined biological and social vulnerabilities and health profiles of various populations [24,29-35]. The biosocial contingencies of pregnancy generally and how these are enhanced in particular settings in SSA constitute a pressing domain of syndemics investigation.

The syndemics of pregnancy in SSA can be categorized for the purposes of analysis into three types: infectious syndemics, mixed infectious/noninfectious syndemics, and noninfectious syndemics. Each of these will be reviewed in turn based on the available literature.

#### Infectious syndemics of pregnancy in Africa

Infectious diseases still cause the majority (69%) of deaths in SSA [36]. Several diseases are of particular note, including HIV, malaria, and tuberculosis (TB).

### Syndemics of HIV disease

Meta-analysis of available global research on the contribution of HIV disease to maternal mortality has identified a pooled attributable risk across studies of 994 per 100,000 women [37]. Of the world’s regions, the contribution of HIV disease to maternal mortality is highest in SSA, accounting for an estimated 207,000 (9%) of deaths between 1990 and 2008 among pregnant and immediate postpartum women [4]. Other estimates suggest pregnancy-related HIV deaths as high as 25% of all maternal deaths for the region [37]. A secondary analysis of pooled community-based data for Sub-Saharan countries by Zaba *et al.*[38] found that excess mortality attributable to HIV was 51.8 (ranging across data sets from 47.8–53.8) per 1,000 person-years in women who were not pregnant or postpartum, and 11.8 (8.4–15.3) per 1,000 person-years in pregnant or postpartum women. These researchers also found that HIV-infected pregnant or postpartum women have a mortality rate that is eight times higher than their HIV-uninfected counterparts. Sub-Saharan countries experiencing increases in maternal deaths in the midst of the campaign to achieve MDG-5, such as Botswana, Swaziland, Lesotho, and South Africa, are all in the subregion of the continent with the highest prevalence of HIV disease. This recognized significant impact of HIV disease on maternal health notwithstanding, there is recognition that “remarkably little is known about the mortality attributable to HIV during the pregnancy and postpartum period [37]”. One area in need of greater understanding is the role of HIV-related disease interactions in maternal illnesses (e.g., anemia, postpartum hemorrhage and puerperal sepsis) and death.

What is known is that HIV disease is highly syndemogenic [15]. A key reason it has become one of the major diseases of human history is its considerable capacity to participate in damaging disease interactions of three types. First, interactions with opportunistic diseases that generally are only able to take hold because of immunodeficiency resulting from HIV disease. Second, interactions with a set of commonly non-opportunistic diseases, such as TB, hepatitis, malaria, and various STDs. Finally, HIV disease interacts adversely with various noninfectious diseases and disorders, including food insufficiency/malnourishment, among others. Each of these will be discussed in turn.

### HIV interaction with opportunistic diseases

There are multiple opportunistic diseases found among HIV-infected pregnant women [39,40]. A study of this population at a large HIV treatment center in Lagos, Nigeria found that the most prevalent opportunistic diseases were oropharyngeal and vaginal candidiasis (24.0% of identified opportunistic disease cases), bacterial infections including pneumonia (12.9%), Herpes zoster (5.3%), and diarrheal diseases (18.1%) [41]. These opportunistic diseases were found to be an important predictor of anemia, a well-recognized risk for heightened morbidity and mortality in pregnant women [42,43]. Analysis of data from the Saving Mothers Report, 2005–2007 by Moran and Moodley [44] found that the most common cause of maternal deaths in known HIV-infected women in South Africa (accounting for 43.7% of all maternal deaths) were nonpregnancy-specific opportunistic diseases such as meningitis and pneumonia. Opportunistic diseases, including meningitis, toxoplasmosis, encephalitis, and pneumonia, were found by Onakewhor and coworkers [45] to be important factors in maternal mortality in Benin City, Nigeria.

An examination was conducted by Djigma *et al.*[46] on the prevalence of bacterial vaginosis—the most common vaginal infection in women of childbearing age—in 251 HIV-positive compared to 200 HIV-negative women at a maternal and child health program in Burkina Faso. They found a range of vaginal pathogens, including several associated with sterility. Nugent’s scores, a Gram-stain scoring system used to diagnose bacterial vaginosis, were significantly higher in HIV-positive women (p < 0.001). As these studies suggest, various opportunistic diseases remain important factors shaping maternal health among HIV-infected women in SSA.

### HIV interaction with non-opportunistic diseases

One of the significant non-opportunistic diseases with which HIV disease increasingly interacts in SSA is TB [47]. While sometimes described as an opportunistic disease, TB has a protracted history as a serious human ailment that long predates the emergence of HIV disease [48]. According to the World Health Organization, maternal TB, which may be harder to detect because of the masking effects of pregnancy, causes a two-fold increase in vaginal bleeding, eclampsia, and pre-eclampsia [49]. Recent research has identified the development of active TB in HIV-infected women in parts of SSA as a grave risk factor for maternal health [50-52]. Because of several pathways of interaction, including HIV reactivation of latent TB and bidirectional disease enhancement involving accelerated progression of both HIV disease and TB, in combination, these two diseases have been shown to have significantly greater impact than the mere addition of their individual effects. This pattern was described by Pillay *et al.*[53] based on a review of existing studies showing that approximately one-sixth of all maternal deaths in referral health centers in southern Africa were associated with TB/HIV coinfection. Further, over one-third (37%) of HIV-infected mothers who were dually infected with TB were severely immunocompromised, with CD4 counts of fewer than 200 cells/microL compared with 14-19% in mothers enrolled in mother-to-child intervention trials in Europe.

Exemplary of TB/HIV coinfection research, Khan *et al.*[54] examined maternal mortality at King Edward III Hospital in Durban, South Africa, located in an area where TB and HIV have emerged as significant entwined contributors to maternal mortality. They found a mortality rate of 323/100,000 among HIV-infected women, compared to 148.6/100,000 among uninfected women. Ninety-three percent (14 of 15) of mothers diagnosed with TB were coinfected with HIV. The hospital-based mortality rate for TB and HIV coinfection was 121.7/1,000; for TB without HIV coinfection, this rate was 38.5/1,000 or about one third the rate of HIV-infected women. Generally, in late HIV disease, women are more likely to have clinical TB, so active TB may be a marker of late HIV disease, as well as an increased risk of death as a result of the comorbidity. Still unresolved in the literature is the issue of pregnancy effects (e.g., added biological and metabolic costs) of TB/HIV interaction, progression, and adverse outcomes.

Another HIV syndemic threat to maternal health involves coinfection with malaria. At early stages in pregnancy, a transient depression of cell-mediated immunity occurs that increases susceptibility to malaria, especially in primagravid and secundigravid women [55]. Independent of HIV, maternal malaria is associated with anemia and maternal mortality [56-58]. It is estimated that 15% of maternal anemia in SSA is linked to malaria and a range of other adverse outcomes [59,60]. It is further estimated that one million pregnancies per year in the region are adversely complicated by coinfection of malaria and HIV disease [61].

Maternal HIV disease is associated with an increased risk of maternal malaria in women of all gravidities in endemic areas, but perhaps not equally so [62]. In a study of women who presented at two rural hospitals in southern Malawi for antenatal care and for delivery, prevalence of HIV disease was 25.6% [63]. In this sample, among women who were pregnant for the first time, prevalence for malaria at enrollment was 56.3% in HIV-infected, and 36.5% in HIV-uninfected, women. For multigravid women, the corresponding rates were 23.8% and 11.0%, respectively. HIV-infected primagravid women exhibited increased malaria prevalence at all gestational stages. The relative risk for malaria infection in HIV-infected women compared to HIV-uninfected women was significantly increased in most parity groups, including women with more than three prior pregnancies (95% CI: 0.31–5.29), suggesting that parity-specific immunity to malaria (found in HIV-negative women) was impaired in dually infected women. Malaria prevalence at delivery remained high in HIV-infected women even among those pharmaceutically treated for infection.

The importance of HIV in malaria infection is further indicated by a study in Kinshasa, Democratic Republic of Congo. In the study, Modia *et al.*[64] compared 146 HIV-infected and 149 HIV-uninfected mothers. Placental biopsies confirmed the presence or absence of placental malaria. These researchers found that the placental malarial infection prevalence was 91% among HIV-infected mothers but only 53.7% in HIV-uninfected mothers (p < 0.0001). Similarly, in a study of 986 pregnant women admitted during the rainy season to the obstetric division of a district hospital in northern Zimbabwe, researchers found that HIV-infected women were more likely to develop malaria attacks—defined as the presence of asexual forms of *Plasmodium* species on a blood smear associated with fear, headache, chills, and/or joint pain—during their pregnancies than women who were seronegative (odds ratio = 3.96, 95% CI: 2.42–6.46) [65]. In a public hospital in western Kenya, HIV-seropositive women coinfected with malaria were twice as likely to suffer from anemia as HIV-seronegative women with or without malaria infection [66]. These findings are of note because under conditions of climate change and extensions in the ranges of malaria vectors, the location and frequency of syndemic interaction of HIV and malaria is likely to increase [67].

Adverse interactions with hepatitis infections, which share common modes of transmission with HIV, constitute another group of non-opportunistic HIV syndemics among pregnant women in SSA. Hepatitis C infection (HCV) is disproportionately common in Africa, which is reported to have the highest prevalence rate (prevalence = 5.3%) of the world’s regions, while the largest reservoirs of chronic hepatitis B (HBV) infection are in SSA (prevalence = ≥8%) and Asia (prevalence = >8%) [68,69]. HIV is known to adversely impact the course of both HBV and HCV infections by accelerating the progression of chronic liver disease. Among individuals previously exposed to HBV in which disease development was contained by the immune system, for example, severe immunosuppression caused by HIV can lead to HBV reactivation [68]. Both types of hepatitis have been identified as risk factors among pregnant women, and are believed to be leading causes of maternal mortality in some SSA nations [70,71]. In a study of HIV/HCV coinfection among 547 pregnant women attending the antenatal clinic of a medical center in Ouagadougou, Burkina Faso, Simpore *et al.*[72] found that 10.6% were positive for HIV disease and 3.3% for HCV disease. Seven women in the sample (1.3%) were dually infected (compared to 76 women with a single infection), a higher than expected rate “suggesting a correlation between these two infections [72]”. A follow-up study of 607 women found higher rates of HCV in HIV-positive women than their HIV-negative counterparts (2.38% versus 1.75%, respectively), but not at statistically significant levels (P = 0.81) [73]. Similarly, a study of pregnant women in Zambia found somewhat higher levels of HBV infection among HIV-positive pregnant women [74].

In a study of over 1,000 pregnant women (half HIV-positive) in Abidjan, Côte d'Ivoire, researchers tested for HBV and HCV infection [75]. They found that HBV was more frequent in HIV-positive women (26.7%), compared to HIV-negative women (9.4%). By contrast, there were no differences in the rates of HCV infection in the two HIV-status groups. A study in Ibadan, Nigeria also found greater coinfection with HBV (8.9%) than HCV (1.9%) in HIV-infected pregnant women [76].

In another study that examined both HBV and HCV among HIV-positive pregnant women in the region, Okeke and co-workers [77] implemented a retrospective survey of approximately 400 HIV-positive pregnant women in Enugu, Nigeria. The combined prevalence of HIV/HBV and HIV/HCV coinfection was 6.5%, with HIV/HBV coinfection being the more common dual infection pattern. Of note, a study of six viral infections among pregnant women (N = 492) and blood donors (N = 191) in rural and urban (Ouagadougou) Burkina Faso found that 0.6% of participants were triply infected, with the most common coinfection array involving HBV, HIV, and human herpesvirus type 8 (HHV-8) [78]. The same trichotomous syndemic was examined in a second Burkina Faso study among over 375 women seeking antenatal consultation at Saint Camille Medical Centre in Ouagadougou [79]. Among HBV-positive patients, HIV and HHV-8 coinfections were high, comprising 16.7% and 20%, respectively, of women who tested positive for hepatitis exposure. Additionally, of the 12.7% of women who were positive for HIV-1, 4.2% also were coinfected with HIV-2. At the same medical center, Ouermi and colleagues (2009) [80] investigated coinfections of HBV and *Toxoplasma gondii—*a parasitic protozoan that causes toxoplasmosis—in HIV-positive and HIV-negative pregnant women. They found that HIV-positive status was associated with higher prevalence rates of both *T. gondii* (31.9 vs. 22.5%, 95% CI: 24.36–40.43) and HBV (13.0 vs. 5.8%, 95% CI: 8.12–20.09).

As these studies suggest, the extent of the HIV disease/hepatitis syndemic, along with other coinfections, among pregnant women in SSA is currently low but higher for HBV/HIV than HCV/HIV coinfection. Changes in risk patterns, such as the growing spread of injection drug use and risky sexual practices among youth and young adults, however, could alter this profile and produce impactful syndemics involving HIV and hepatitis [81,82].

### HIV interaction with noninfectious diseases and disorders

The third group of diseases and health conditions that HIV disease interacts with includes various noninfectious diseases and disorders, such as food insufficiency [83-85]. These terms are defined as persistent lack of access to adequate food in needed quantity and quality (food insecurity), and deficiencies in micronutrients and macronutrients (undernutrition) [86]. A mother’s nutritional status is a critical determinant of maternal health [87]. In SSA, HIV disease has spread rapidly among populations in which malnutrition is endemic. Syndemic interaction between these two health threats is centered in the body’s complex and multilayered host immune system [88]. In fact, malnutrition and HIV disease are the two most common causes of acquired immune dysfunction, and the patterning of immune system suppression caused by malnutrition mirrors, in many ways, the downgraded immune effects of HIV disease. The pathways of interaction are bidirectional. While HIV disease exacerbates nutrient deficiencies by altering critical metabolic processes involved in nutrient intake and utilization, chronic undernutrition weakens the body’s immune response, leading to reductions in immune cell populations and immunocompetence. These dynamics have been identified as a significant threat to pregnant women in SSA [89-92].

Beyond HIV disease, a range of other infectious syndemics impacts the health of pregnant women in SSA. One of these noteworthy in the region is the interaction of malaria and helminth infections.

### Malaria and helminths

The conditions for a potential syndemic of these two parasitic infections are created by the overlapping geographic distributions of mosquito vectors of malaria and the various species of intestinal helminths. An assessment of over 1,000 febrile patients in southern Ethiopia found that malaria infection was more common in patients coinfected with the helminths *A. lumbricoides* (21.3%), *T. trichiura* (23.1%), and *S. mansoni* (23.1%) than those patients without a helminth infection (9.3%) [93]. The prevalence of non-severe malaria was significantly higher in individuals infected with specific helminths (*A. lumbricoides*-35.9%, *T. trichiura-*11.7%, *S. mansoni*-9.8%, and hookworm-9.8%, respectively) than those who were not so infected (9.3%) (adjusted OR = 1.58, 95% CI: 1.13–2.22). Moreover, the odds ratio of having non-severe malaria increased with the number of intestinal helminth species infecting a patient (p < 0.001). Coinfected individuals showed lower mean levels of hemoglobin than those with malaria infection alone (p = 0.027). The specific helminth species involved may be critical, as some findings suggest a protective or counter-syndemic effect with particular types of intestinal worms and a worsening syndemic effect with others [94]. Hookworm, for example, falls into the latter group, which is consequential both because its prevalence in SSA among pregnant women is high and because it “may contribute significantly to the degree of anemia in pregnant women [95]”.

Several hypotheses have been put forward to explain the nature of the interaction between helminths and malaria. A review by Mwangi *et al.*[96] highlights the possibility that helminth infection creates a cytokine milieu that is favorable to the production of non-cytophilic antibodies, making individuals more susceptible to clinical malaria. Alternately, Yazdanbakhsh *et al.*[97] suggest that the presence of T-regulatory cells increase during helminth infection, which, if present in sufficient numbers, induce a non-specific immune suppression that facilitates malaria development upon exposure. As yet, given the limited availability of studies, the relative value of these hypotheses remains uncertain [98].

First described over 75 years ago, it is clear that dual infection of malaria and intestinal helminths presents a serious threat to the health of pregnant women in SSA [99]. Varying patterns of coinfection have been described. A cross-sectional study of women presenting for delivery at two hospitals in Kumasi, Ghana, found that 19.7% were positive for the malarial pathogen *P. falciparum*, 9.1% were positive for helminth infection without malaria, and 16.6% were coinfected [100]. Several helminths were identified, including hookworms, *A. lumbricoides*, *T. trichiura*, and *S. stercoralis*, with the first two being the most common. Women with intestinal helminths were almost five times as likely to be infected with malaria as women without a helminth infection. Young age at pregnancy was found to be strongly associated with dual infection, while heightened rates of dual infection also were found among single, low-income, and primagravid women. Hiller *et al.*[101] observed “a strong association between asymptomatic infection with *P. falciparum* and infection with [the helminth] *M. perstans*” in a randomized, double-blind, placebo-controlled treatment study among pregnant women in Entebbe, Uganda. A weaker association was observed between hookworm and *P. falciparum infection*. Egwunyenga *et al.*[95] randomly selected over 2,000 near-term pregnant women who delivered at three hospitals in Nigeria for screening for malaria and helminth parasites. Their study revealed that over 45% of *Plasmodium* infected women also harbored intestinal helminths. Women, especially primagravids, with *Plasmodium*/intestinal helminth coinfections had lower hemoglobin levels than those who suffered from malaria infection alone. This condition was attributed to chronic loss of blood and iron due to both infections. Anemia was believed to be further aggravated by the poor nutritional status of study participants, especially involving limited access to folate and iron.

Taking the assessment of disease interaction another step, Crowther *et al.*[102] examined the interplay of malaria, helminths, and HIV disease among 328 HIV-positive women attending antenatal centers in Rwanda. They found that 38% tested positive for helminths, 21% had malaria, and 10% had dual infection. The most prevalent helminth was *A. lumbricoides* (20.7%), followed by *T. trichiura* (9.2%). Women with helminth infections were characterized by low hemoglobin and CD4 counts (p < 0.05). This study demonstrates the importance of assessing multi-disease syndemics among pregnant women. In low-income settings in particular, pregnant women may be subject to complex syndemics involving more than two diseases in adverse interaction.

#### Mixed infectious/noninfectious syndemics

Syndemics, as indicated, are not limited to pathogen-pathogen interactions involving infectious diseases, but also include adverse interplay of infectious and noninfectious diseases. It has been established that some infections increase the risk of certain noninfectious diseases and vice versa. Among pregnant women in SSA, this is seen in the TB/diabetes syndemic. Diabetes considerably increases risk of early mortality due to TB [103]. This association may be produced both through the role of diabetes in impairing immune functions and through its interference with the effectiveness of pharmaceutical treatment of TB [104]. Bidirectional impact, involving TB increasing the risk of diabetes, also has been described [15]. Dietary deficiencies, including inadequacies of vitamins A, C, and D, have been linked to increased risk for both diabetes and TB [104]. In SSA, diabetes has been associated with a three-fold incident risk of TB and increased prevalence of diabetes has been identified in patients with pulmonary TB [105-107]. The precise impact of the convergence of these two diseases among pregnant women in SSA, which is expected given the rising prevalence of diabetes and the existing prevalence of TB, remains uncertain because as Dooley and Chaisson [108] found in review of the relevant literature: “Many important topics have been poorly studied or not studied at all”.

#### Noninfectious syndemics

Noninfectious syndemics, involving interaction among chronic diseases and health conditions, are also of critical importance. In the case of pregnant women in SSA, this is seen in the interaction of diabetes and undernutrition. Diabetes contributes to anemia during pregnancy and maternal morbidity [109,110]. Prevalence rates of gestational Type 2 diabetes are increasing globally including in SSA [111]. Gestational diabetes (GDM) is associated with an increased risk for the subsequent development of classical diabetes mellitus. While studies of GDM are limited in African countries [110], one of the highest rates (3.7%) has been found among rural pregnant women in northern Ethiopia [112]. In accounting for this level of prevalence, the authors suggest the importance of nutritional deficiency: “The only possible reason found to be a cause for the high prevalence of GDM in this area is the exposure of these pregnant women to chronic malnutrition during their intrauterine life and early child hood periods. Drought, famine and war have persistently affected this area where the study was conducted for the last over 50 years [112]”. In other research, marginal food security has been found to be significantly associated with GDM [113].

## Conclusion

Pregnant women, particularly in SSA, are an understudied group, but they are more vulnerable to infections because of suppression of the immune system during pregnancy and also are at heightened risk for various noninfectious diseases as well. While amplified vulnerability is recognized, epidemiological, biomedical, and social science of health research about pregnant women has tended to be characterized by “single disease” approaches. In this paper, I have reviewed available literature suggesting the critical importance of adverse disease interactions of infectious and noninfectious diseases to draw attention to the role of syndemics in maternal health in the region.

The strength of the findings of this review are limited by several factors, including the size and significant diversity of the countries and locales of SSA, the burdens of research common to resource-poor settings, the comparatively few studies focused specifically on maternal health in the region, the dominance of narrowly-focused prevalence studies, the small number of ethnographic assessments that present the experience and perspective of pregnant women on their health, and the restricted attention paid to comorbidity, let alone to the nature, pathways and consequences, of disease interaction. Nonetheless, the available literature is adequate to suggest the salience of the syndemics of pregnancy in the countries under examination. Capacity-strengthening efforts designed to improve the number and quality of syndemic “burden-of-disease” studies could provide critical knowledge for the implementation of programs capable of improving the quality of care and maternal health in SSA. Such studies would explore the constellation of diseases of pregnant patients; would investigate locally-identified syndemics and their health effects during pregnancy including the patterns of presentation of specific interacting diseases, the social conditions that foster disease clustering and interaction among pregnant women; and involve trials of integrated treatment regimens designed to treat multiple co-occurring diseases in this population.

Ray and co-authors [114] argue that “failure to reduce preventable maternal deaths represents a violation of women’s right to life, health, non-discrimination and equality.” They advocate an activist approach, involving collaboration of health professionals and non-government civil organizations for improving material health in the African context. As a model for such an initiative, they point to grassroots HIV activism and its successful rights-based challenge. Thus, they note: “With regard to maternal mortality, where the majority of deaths are preventable and in many countries occur in health institutions, there are opportunities to learn from the successes of HIV campaigns in making health services more responsible to women’s needs [114]”. As an example of this kind of activism in women’s health, they point to the Treatment Action Campaign in South Africa, which used a right to health approach, community empowerment, popular mobilization, and legal action. The syndemics model, which emphasizes the social origins of disease and the need for health care response to the entwined biosocial complexities of living conditions, gender discrimination, pregnancy under conditions of poverty, and the role of a constellation of interacting diseases can inform activist efforts to improve maternal health in SSA.

## Abbreviations

GDM: Gestational diabetes; HBV: Hepatitis B virus; HCV: Hepatitis C virus; HIV: Human immunodeficiency virus; MDGs: Millennium development goals; SSA: Sub-Saharan Africa; TB: Tuberculosis.

## Competing interests

The author declares that he has no competing interests.

## Acknowledgement

The author thanks Balya Ostrach for her suggested revisions based on an earlier draft of this paper.

## Supplementary Material

Additional file 1 Multilingual abstracts in the six official working languages of the United Nations. Click here for file
