# Supplementary material for: Development, coinfection, and the syndemics of pregnancy in Sub-Saharan Africa
Source: Infect Dis Poverty. 2013 Nov 15;2:26. doi: 10.1186/2049-9957-2-26 (PMC4177213; doi:10.1186/2049-9957-2-26)
Supplement: Additional file 1 — Multilingual abstracts in the six official working languages of the United Nations. [file 2049-9957-2-26-S1.pdf]

Translation of the abstract into the six official working languages of the United Nations

التنمية والعدوى مُصاحبة والعدوى الجماعية أثناء الحمل في جنوب الصحراء الكبرى في إفريقيا

ميريل سنجر

الملخص

إنّ من أبرز الثغرات لتحقيق الأهداف التنموية للألفية في مجال الصحة العالمية (MDG) هي أوجه القصور في مُعالجة صحة الأمهات، وهي مسألة قد تناولتها الأهداف التنموية للألفية الخامسة. كما أنّ هذا النقص الملموس بشكلٍ حادٍ في جنوب الصحراء الكبرى في إفريقيا (SSA)، حيث تحدث أكثر من نصف إجمالي عدد وفيات الأمهات كل عام. في حين أنه لا يُوجد هناك حتى الآن فهماً شاملاً للأسباب البيولوجيـة والاجتماعية لوفيات الأمهـات في جنوب الصحراء الكبرى في إفريقيا، فمن الواضح أن شدة الفقر والتهميش الاقتصادي، والاضطرابات الاجتماعية، وصعوبات توفير الرعاية الصحية، والتفاوت في نوعية الرعاية الصحية، وعمليات الإجهاض غير القانونية والسرية، والالتهابات، كلها عوامل حاسمة. وبعيداً عن هذه العوامل، تعرض هذه الورقة البحثية سرداً متكاملاً للدراسات السابقة والتي تتناول صحة الأم في جنوب الصحراء الكبرى في إفريقيا وتُناقش قضية العدوى الجماعية التي تُشكل مصدراً إضافياً ضخماً في أمراض ووفيات الأمهات في فترة النفاس في تلك المنطقة. ونتيجة لذلك، تُؤكد الورقة على أهمية زيادة التركيز على الطبيعة، والوقاية، والعلاج من عدوى الإلتهابات الجماعية، والتي ينبغي أن تكون جزءاً لا يتجزأ من تحسين صحة الأمهات في جنوب الصحراء الكبرى في إفريقيا.

Translated from English version into Arabic by Freelanceabunar, through

TRANSLATORS
WITHOUT BORDERS

撒哈拉沙漠以南非洲地区孕产妇的卫生保健，混合感染和共存疾病

Merrill Singer

摘要

改善孕产妇保健被列为第五项联合国千年发展目标，是联合国千年发展规划面临的主要挑战之一。孕产妇保健问题在撒哈拉沙漠以南非洲地区尤为严峻。据统计，全球每年有逾半数的孕产妇死于该地区。虽然目前在生物学和社会学方面尚未取得足够的进展，但是显然，孕产妇死亡与贫穷、因性别差异导致的经济边缘化以及社会动荡等因素是分不开的，这些因素阻碍了孕产妇卫生保健的可及性；除此之外，医疗卫生服务不平等，违法和非法堕胎，以及传染病也是至关重要的因素。本文综述了撒哈拉沙漠以南非洲地区孕产妇健康问题，并讨论了共存疾病是否是该地区孕产妇发病和死亡的一个要素。科学研究、预防与治疗共存疾病应成为撒哈拉沙漠以南非洲地区提高孕产妇健康水平的重要内容之一。

Translated from English version into Chinese by Qian Ying-jun, through

CDC
CHINESE CENTER FOR DISEASE CONTROL AND PREVENTION
NATIONAL INSTITUTE FOR PARASITIC DISEASES

Le développement, la co-infection et les syndemics de la grossesse en Afrique subsaharienne.

Merrill Singer

Résumé

Parmi les lacunes notables dans la réalisation des Objectifs du millénaire pour le développement (OMD) figurent les lacunes dans le traitement des questions de santé maternelle, une question faisant l'objet du cinquième OMD. Cette lacune est particulièrement grave en Afrique subsaharienne (ASS) où plus de la moitié de tous les décès maternels surviennent chaque année. Bien qu'il n'y ait pas encore de compréhension globale des causes sociales et biologiques des décès maternels en ASS, il est évident que la pauvreté, la marginalisation économique basée selon le genre, les perturbations sociales, les difficultés d'accès aux soins, les disparités dans la qualité des soins, les avortements clandestins et illégaux ainsi que les infections sont tous des facteurs critiques. Au-delà de ces facteurs, cet article présente un aperçu de la littérature existante sur la santé maternelle en ASS pour soutenir que les syndemics constituent une source supplémentaire de morbidité des mères et de mortalité dans la région. Porter un intérêt croissant à la nature, à la prévention et au traitement des syndemics devrait par conséquent faire partie intégrante de l'amélioration de la santé maternelle en ASS.

Translated from English version into French by Laetitia Michel, through

TRANSLATORS
WITHOUT BORDERS

Развитие, конинфицирование и синдемия беременных женщин в Центральной и Западной Африке

Мэррилл Сингер

Резюме

Цели развития тысячелетия (ЦРТ) содержат ряд важных пробелов, среди которых можно назвать недостаток внимания к вопросу здоровья матери — проблеме, которой посвящена пятая ЦРТ. Особенно это касается региона Центральной и Западной Африки, на который приходится свыше половины всех случаев материнской смертности в мире ежегодно. Хотя пока до конца непонятно, каковы социальные и биологические причины материнской смертности в данном регионе, вполне очевидно, что ключевые факторы включают бедность, гендерную социально-экономическую изоляцию, социальные волнения, затрудненный доступ к медицинскому обслуживанию и различия в его качестве, незаконные и подпольные аборты и инфекции. Помимо указанных факторов, в этой работе представлен обзор существующей литературы в сфере здоровья матери в Центральной и Западной Африке, подтверждающей факт того, что синдемия является немаловажной дополнительной причиной материнской смертности в регионе. Повышенное внимание к природе, предупреждению и преодолению синдемии должно стать неотъемлемой частью роста уровня материнского здоровья в Центральной и Западной Африке.

Translated from English version into Russian by Irina Zayonchkovskaya, through

TRANSLATORS
WITHOUT BORDERS

Desarrollo de enfermedades, coinfección y sindemia durante el embarazo en el África subsahariana

Merrill Singer

Resumen

Una de las diferencias más notables entre los logros conseguidos gracias a los Objetivos de Desarrollo del Milenio (ODM) son las deficiencias a la hora de gestionar la salud materna, cuestión que se planteó en el quinto ODM. Esta deficiencia es especialmente grave en el África subsahariana (ASS), donde cada año se producen más de la mitad de las muertes maternas de todo el planeta. Aunque siguen sin entenderse del todo las causas sociales y biológicas de la muerte materna en el ASS, resulta evidente que la pobreza, la marginación económica y de género, los trastornos sociales, el limitado acceso a la sanidad y las desigualdades en su calidad, los abortos ilegales y clandestinos y las infecciones son, todos ellos, factores críticos. Además de estos factores, este artículo presenta un análisis de la bibliografía sobre salud materna en el ASS con el objeto de argumentar que la sindemia constituye una importante fuente de enfermedad y muerte materna en la región. Por todo lo anterior, la sanidad materna en el ASS debería incrementar sus esfuerzos en la naturaleza, prevención y tratamiento de la sindemia.

Translated from English version into Spanish by Rosa Sanz, through

TRANSLATORS
WITHOUT BORDERS
